# Lower cortical thickness and increased brain aging in adults with cocaine use disorder

**DOI:** 10.3389/fpsyt.2023.1266770

**Published:** 2023-11-13

**Authors:** David Schinz, Benita Schmitz-Koep, Marlene Tahedl, Timo Teckenberg, Vivian Schultz, Julia Schulz, Claus Zimmer, Christian Sorg, Christian Gaser, Dennis M. Hedderich

**Affiliations:** ^1^Department of Neuroradiology, School of Medicine, Technical University of Munich, Munich, Germany; ^2^TUM-NIC Neuroimaging Center, School of Medicine, Technical University of Munich, Munich, Germany; ^3^Institute of Radiology, University Hospital Erlangen, Friedrich-Alexander-Universität Erlangen- (FAU), Nürnberg, Germany; ^4^Digital Management & Transformation, SRH Fernhochschule - The Mobile University, Riedlingen, Germany; ^5^Department of Psychiatry, School of Medicine, Technical University of Munich, Munich, Germany; ^6^Department of Psychiatry and Psychotherapy, Jena University Hospital, Jena, Germany; ^7^Department of Neurology, Jena University Hospital, Jena, Germany; ^8^German Center for Mental Health (DZPG), Site Jena-Magdeburg-Halle, Germany; ^9^Center for Intervention and Research on Adaptive and Maladaptive Brain Circuits Underlying Mental Health (C-I-R-C), Jena-Magdeburg-Halle, Germany

**Keywords:** BrainAGE, cortical thickness, cocaine, substance use disorder, addiction, aging, cocaine use disorder

## Abstract

**Background:**

Cocaine use disorder (CUD) is a global health issue with severe behavioral and cognitive sequelae. While previous evidence suggests a variety of structural and age-related brain changes in CUD, the impact on both, cortical thickness and brain age measures remains unclear.

**Methods:**

Derived from a publicly available data set (SUDMEX_CONN), 74 CUD patients and 62 matched healthy controls underwent brain MRI and behavioral-clinical assessment. We determined cortical thickness by surface-based morphometry using CAT12 and Brain Age Gap Estimate (BrainAGE) via relevance vector regression. Associations between structural brain changes and behavioral-clinical variables of patients with CUD were investigated by correlation analyses.

**Results:**

We found significantly lower cortical thickness in bilateral prefrontal cortices, posterior cingulate cortices, and the temporoparietal junction and significantly increased BrainAGE in patients with CUD [mean (SD) = 1.97 (±3.53)] compared to healthy controls (*p* < 0.001, Cohen’s *d* = 0.58). Increased BrainAGE was associated with longer cocaine abuse duration.

**Conclusion:**

Results demonstrate structural brain abnormalities in CUD, particularly lower cortical thickness in association cortices and dose-dependent, increased brain age.

## 1. Introduction

Cocaine use disorder (CUD) is part of a spectrum of substance use disorders (SUD) that, according to the United Nations Office on Drugs and Crimes’ World Drug Report 2022, imposes a global health issue with approximately 21.5 million cocaine users annually, which translates into a 0.4% prevalence in the population aged 15–64. The prevalence of cocaine dependence, the second most commonly used illicit drug in Europe (1), is estimated to be approximately 1–2% in both European and American populations, with significantly more males affected (2, 3). CUD is defined in the DSM-5 as a specific pattern of cocaine use that leads to significant clinical impairment or distress and is caused by genetic, environmental, biological, and psychological factors (4). Behavioral-cognitive changes in CUD include changes in attention, compulsivity, impulsivity, and memory, thus making underlying brain changes plausible (5, 6).

In fact, frequent cocaine use is associated with microstructural, macrostructural, and functional brain changes, most of which have been demonstrated in animal studies (7–10). In humans, there is less and inconsistent evidence of structural changes in cocaine addicts, with most studies showing volumetric changes in prefrontal regions (9, 11–18). These structural changes have been shown to correlate with behavioral and cognitive measures such as impulsivity and compulsivity (19). However, it is unclear which distinct brain aspects contribute to the volume changes, such as cortical thickness. Therefore, the current study focuses on cortical thickness in CUD. We chose surface-based morphometry to directly assess cortical thickness as a distinct marker of brain integrity and cortical atrophy (20–22). Furthermore, frontal grey matter volume losses in patients with CUD compared with healthy controls (HC) have been shown to be age-related, suggesting potentially accelerated brain aging in CUD (23). An established biomarker of accelerated brain aging that is also related to cortical thickness, is the brain age gap estimation (BrainAGE) (24). BrainAGE is measured by structural MRI combined with machine-learning algorithms to estimate the difference between brain measures of a normal population and an index group (25–27). This biomarker has been reliably used to calculate the offset between biological and chronological brain age based on MRI and identified significant differences in various neuropsychiatric disorders (28–30). The current study therefore also focuses on BrainAGE in CUD.

In this study, we investigated the impact of CUD on biological brain age and cortical thickness in a relatively large cohort of adults with CUD and matched HC. We hypothesized that patients with CUD would have a significantly accelerated biological brain age and lower cortical thickness in prefrontal cortices (13, 14). To test these hypotheses, we analyzed high-resolution structural 3D T1-weighted MR images using surface-based morphometry and relevance vector regression. In addition, we investigated possible associations of potential changes in both accelerated biological brain age and cortical thickness with behavioral-clinical assessments, such as impulsivity and compulsivity, in the CUD cohort. Due to previously described widespread brain changes in CUD, we expected particularly that global brain age changes might be linked with both the ‘dose-effect’ and the most relevant behavioral changes of cocaine use, such as craving or impulsivity.

## 2. Materials and methods

### 2.1. Participants

A detailed description of the evaluated cross-sectional Mexican Substance Use Disorder neuroimaging dataset (SUDMEX_CONN) can be found in: (9, 31, 32). Briefly, 145 participants (patients with CUD and HC between 18 and 50 years of age) were enrolled in the study between March 2015 and October 2016. Cocaine dependence was diagnosed in patients with CUD using the MINI International Neuropsychiatric Interview - Plus Spanish version 5.0.0 (33) administered by trained psychiatrists.

Patients with CUD were actively consuming cocaine or had been abstinent for less than 60 days prior to the study; frequency of use had to be at least 3 days per week; and abstinence had to be no more than 60 days continuously in the past 12 months. Additional inclusion/exclusion criteria have been published with the dataset or have been made available at: https://zenodo.org/record/5123331. Verbal and written informed consent were obtained from all participants. The study was conducted in accordance with the Declaration of Helsinki and approved by the local ethics committees of the Instituto Nacional de Psiquiatría “Ramón de la Fuente Muñiz” in Mexico City, Mexico. Participants were invited through posters placed in addiction treatment centers.

Of the total 145 participants, 7 were excluded from data collection (see Supplementary material). In addition, 2 participants (HC) were excluded due to missing demographic data (age and sex). Thus, the final sample consisted of 136 individuals [74 patients with CUD (9 females) and 62 HC (11 females)].

### 2.2. Clinical and behavioral assessments

In the SUDMEX-CONN study, clinical standardized questionnaires, medical interviews, and cognitive tests were completed by participants. The tests were administered by trained psychiatrists and psychologists. We focused on the most prominent behavioral measures in CUD, namely craving as assessed by the Cocaine Craving Questionnaire (CCQ), which measures patients’ drug-related craving within the past week and has been validated in the Mexican population (34, 35), and impulsivity as assessed by the Spanish translation of the Barratt Impulsiveness Scale version 11 (BIS), that measures impulsive behaviors by the 3 categories of attentional, motor, and non-planning impulsiveness (36, 37). In addition, we assessed executive functioning by the Berg’s Card Sorting Test (BCST) (38, 39). Furthermore, we the average weekly cocaine dose and years consuming cocaine as a proxy for the cocaine dependent ‘dose-effect’.

### 2.3. Imaging data acquisition

MRI data were obtained using a Philips Ingenia 3 Tesla MR system (Philips Healthcare, Best, The Netherlands, and Boston, MA, United States), with a 32-channel dS Head coil. Breath alcohol tests were performed before MRI acquisition. High-resolution structural T1-weighted (T1w), 3D-FFE SENSE sequence was performed with the following parameters: TR/TE = 7/3.5 ms, field of view = 240 mm^2^, matrix = 240 × 240 mm, number of slices = 180, gap = 0, plane = sagittal, voxel = 1 × 1 × 1 mm (the first five participants were imaged with a voxel size of 0.75 × 0.75 × 1 mm). Image data quality was assessed visually and quantitively using MRIQC v. 0.15, which is available on the OpenNeuro platform and can be found in the dataset release publication. All images passed homogeneity control as implemented in the CAT12 toolbox (40).

### 2.4. MRI preprocessing

MRI data were processed in Matlab 2021b^1^ using SPM12^2^ and the CAT12 toolbox^3^ (40). As detailed elsewhere, (26) all images were corrected for magnetic field inhomogeneities, tissue-classified into gray matter, white matter, and cerebrospinal fluid, and spatially normalized to MNI space using 12-parameter affine transformations. The normalized gray and white matter segments were then smoothed using a 4 mm and 8 mm FWHM Gaussian Kernel, and the images were resampled to 4 mm and 8 mm. These processed images provided the input for the BrainAGE analysis.

### 2.5. BrainAGE estimation

The BrainAGE framework is based on a Relevance Vector Regression Machine (RVR) (41). Based on T1-weighted images it models healthy brain development and aging, allowing individual brain age estimation (26). The initial model was trained using chronological age and processed gray and white matter segments based on T1-weighted images of MRI data from the IXI dataset^4^ to extract the salient features of healthy brain development and aging. Depending on the relative importance of each voxel for healthy brain aging voxel-specific weights are calculated. For a detailed description of the most important features that were used by the RVR to quantify healthy brain aging please refer to Franke et al. (26). Finally, individual brain age of a test subject can be calculated based on preprocessed T1-weighted MRI data across the whole brain applying the regression pattern of healthy brain aging. The difference between this estimated and chronological age is the individual’s BrainAGE score. Positive values indicate accelerated and negative values indicate decelerated structural brain aging. The model’s reliability has been demonstrated in several recent works at a mean absolute error of 3.322 years and has been shown to yield intraclass correlation coefficients of 0.9 calculated from two shortly delayed scans on the same MRI scanner (25, 42).

In this study, to consider the age range of our sample we limited the age range of the used training data to 18–50 years and finally used 255 healthy subjects (125 females, mean age 32.6, SD 7.21 years) for training. We applied this trained algorithm to the processed gray and white matter images normalized to MNI space of the current sample to estimate individual BrainAGE. The 8 different models (4 mm and 8 mm for resampling and smoothing, gray and white matter) were combined to one single BrainAGE value using a general linear model that maximized BrainAGE differences between the two groups.

### 2.6. Surface-based morphometry

Surface-based morphometry was also processed using the CAT12 toolbox. The extracted cortical thickness measures were smoothed using a 12 mm FWHM Gaussian Kernel. Surface region of interest (ROI) analysis was performed for 70 ROIs based on the Desikan-Killiany Atlas included in CAT12 (43). A list of the 70 ROIs can be found in the Supplementary material.

### 2.7. Statistical analysis

Statistical analyses on scalar variables were performed using IBM SPSS Version 27 (IBM Corp., Armonk, NY, United States): Normal distribution was assessed with the Shapiro–Wilk test. Differences in clinical variables between patients with CUD and HC were tested with the Chi^2^ test (sex) and two-sample *t*-tests (BCST, BIS). The threshold for statistical significance was set at *p* < 0.05.

#### 2.7.1. Group comparison for cortical thickness

A general linear model (GLM) analysis was used to test for group differences (patients with CUD vs. HC) in cortical thickness of ROIs using the CAT12 toolbox. Sex and age were entered as covariates-of-no-interest. The threshold for statistical significance was set at *p* < 0.05, false discovery rate-corrected (FDRc) (44).

To validate the ROI-GLM approach, we performed vertex-wise GLM analysis to test for group differences (patients with CUD vs. HC) in cortical thickness using the CAT12 toolbox. Sex and age were entered as covariates-of-no-interest. Additional threshold-free cluster enhancement was performed using the TFCE toolbox of CAT12 with 10,000 permutations (45). The threshold for statistical significance was set at *p* < 0.05, family-wise error corrected (FWEc). We did not use total intracranial volume as a covariate-of-no-interest, since cortical thickness is widely independent of head size (46, 47).

#### 2.7.2. Group comparison for BrainAGE

Group difference in BrainAGE between patients with CUD and HC was assessed by one-tailed *t*-test. The threshold for statistical significance was set at *p* < 0.05. Cohen’s d was calculated for group differences.

#### 2.7.3. Linking BrainAGE and behavioral-clinical variables and the ‘dose-effect’

Linkage of BrainAGE to behavioral-clinical variables through statistical analyses was performed using IBM SPSS Version 27 (IBM Corp., Armonk, NY, United States) and SPM12, running under MATLAB 2021b. Correlations between behavioral-clinical variables and individual BrainAGE scores were tested in the CUD cohort with two-tailed partial Spearman correlation analysis, corrected for age and sex, and correlations between the ‘dose-effect’ and individual BrainAGE scores were tested in the CUD cohort with one-tailed partial Spearman correlation analysis, corrected for age and sex. Statistical significance was set at *p* < 0.05. We analyzed the CCQ and BIS scores for cocaine-related behavioral effects, the BCST for cognition, and the years consuming cocaine and average weekly dose of cocaine for cocaine dose-dependent effects on BrainAGE scores.

We also performed these analyses for the regional cortical thickness changes and behavioral-clinical variables, but as the effects of cocaine use on the brain are expected to be widespread, as an exploratory analysis.

Three patients with CUD had missing CCQ-scores, nine patients with CUD had missing ‘dose-effect’ data, and 10 patients with CUD had missing BCST and BIS scores. Subjects were excluded from the respective analyses.

## 3. Results

### 3.1. Sample characteristics

The demographic and clinical characteristics of the study cohort are shown in Table 1. There was no significant difference between patients with CUD and HC in age at scanning (*p* = 0.77) and sex (*p* = 0.54). Patients with CUD made significantly more errors in the BCST (*p* = 0.003) and showed higher impulsiveness in the BIS (*p* < 0.001). Median average weekly dose in grams of cocaine in the past year was 1–4 grams. The average years of consuming cocaine was 9.1 ± 6.8 years.

**Table 1:** Demographical, cognitive, and clinical data of the study cohort.

	Patients with CUD (*n* = 74)			HC (*n* = 62)			
	Mean	SD		Mean	SD		*p*-value
Sex (male/female)	65/9			51/11			0.54
Age (years)	31.0	± 7.3		30.6	± 8.3		0.77
BCST (total errors)^†^	52.6	± 19.2		40.7	± 19.9		0.003
CCQ (*n* = 71)	142.1	±44.2		N/A			
BIS^†^	61.2	±15.4		45.0	±14.9		<0.001
Years consuming cocaine (*n* = 68)	9.1	±6.8		N/A			
Average weekly cocaine dose (*n* = 69)^‡^	1–4 grams			N/A			

^†^, 64 patients with CUD. ^‡^, median.

BCST, Berg’s card sorting test; CCQ, cocaine craving questionnaire, CUD, cocaine use disorder; HC, healthy controls; N/A, not applicable; SD, standard deviation.

### 3.2. Cortical thickness

#### 3.2.1. Reduced cortical thickness in patients with CUD

**Figure 1:**
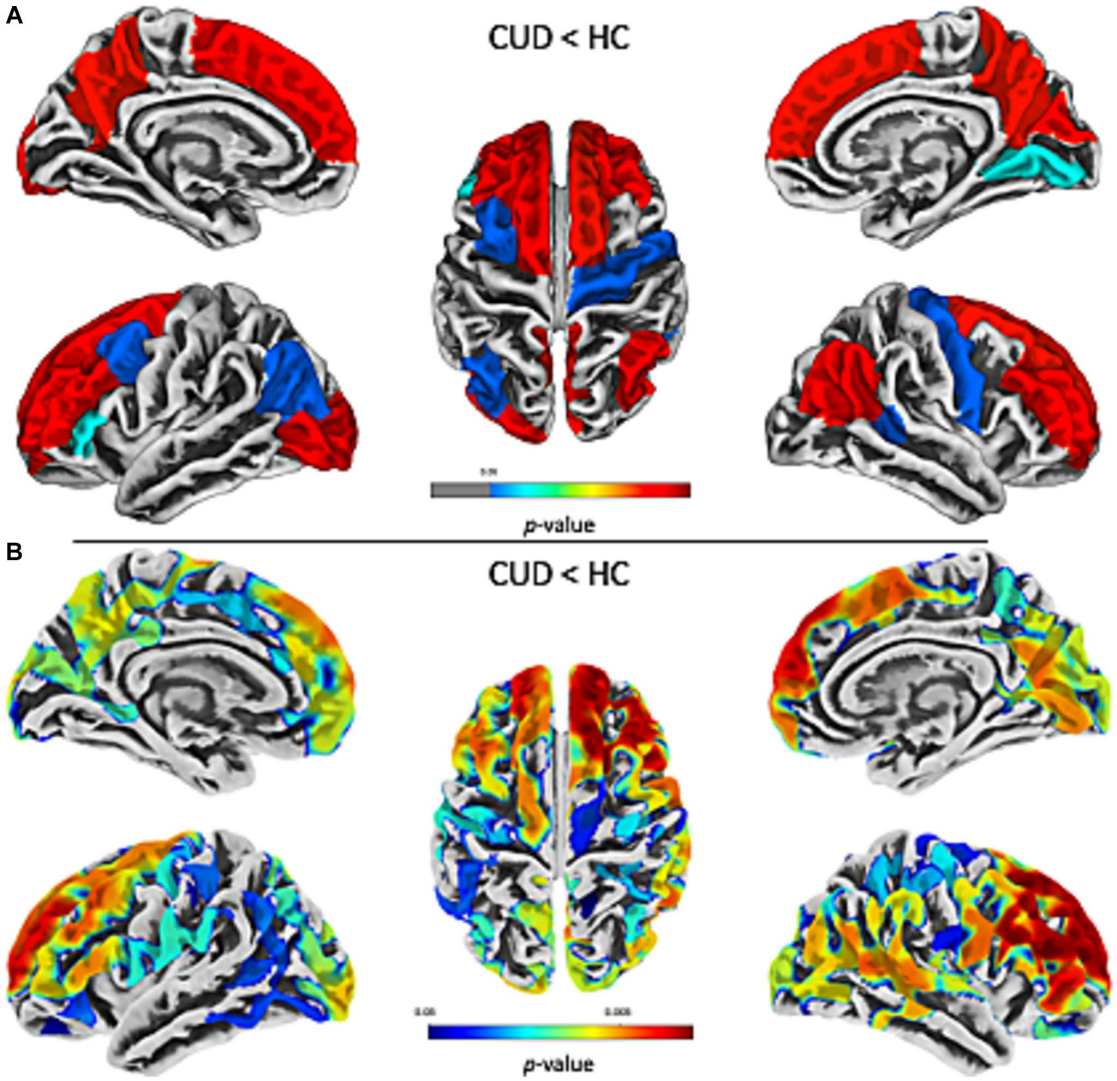
Decreased cortical thickness in prefrontal, temporoparietal, and posterior cingulate association cortices in CUD. The results of the ROI-based **(A)** and vertex-wise **(B)** comparison of cortical thickness in patients with CUD compared with HC show widespread, bilateral reductions of cortical thickness in the prefrontal cortices (superior frontal gyrus and rostral middle frontal gyrus), posterior cingulate cortex, and temporoparietal junction in the CUD cohort. Differences were more pronounced in the right hemisphere [8 (right) and 7 (left) significantly reduced ROIs]. There were no ROIs with a significantly increased cortical thickness in patients with CUD compared to HC. Statistical significance was set at *p* < 0.05, FDRc for the ROI-based approach and *p* < 0.05, FWEc for the vertex-wise approach. Warmer colors indicate lower value of *p*s. CUD, cocaine use disorder; HC, healthy controls; ROI, region of interest.

The results of the ROI-based GLM cortical thickness analysis of patients with CUD and HC showed a significant (*p* < 0.05, FDRc) bilateral reduction of cortical thickness in the prefrontal cortices (superior frontal gyrus and rostral middle frontal gyrus), posterior cingulate cortex and temporoparietal junction in the CUD cohort (Figure 1A). The differences were more pronounced in the right hemisphere [8 (right) and 7 (left) significantly reduced ROIs, see Table 2]. There were no ROIs with a significantly increased cortical thickness in patients with CUD compared to HC.

**Table 2:** ROIs with significantly reduced cortical thickness in patients with CUD compared to HC with *T* and *p* values, FDRc.

ROI	*T*-value	*p*-value
Left hemisphere		
Lateral occipital cortex	2.97	0.026
Superior frontal gyrus	2.92	0.026
Rostral middle frontal gyrus	2.91	0.026
Precuneus cortex	2.76	0.027
Pars triangularis	2.52	0.040
Inferior parietal cortex	2.39	0.049
Caudal middle frontal gyrus	2.35	0.049
Right hemisphere		
Rostral middle frontal gyrus	3.06	0.026
Inferior parietal cortex	2.97	0.026
Cuneus cortex	2.90	0.026
Superior frontal gyrus	2.82	0.027
Precuneus cortex	2.76	0.027
Lingual gyrus	2.54	0.042
Precentral gyrus	2.34	0.049
Banks superior temporal sulcus	2.34	0.049

FDRc, false-discovery rate corrected; ROI, region of interest.

To validate the ROI-based GLM approach, we performed vertex-wise GLM analysis to test for significant cortical thickness group differences in patients with CUD compared to HC. Consistent with the previous approach, we found significant (*p* < 0.05, FWEc) bilateral cortical thickness reductions primarily in the prefrontal gyri (Figure 1B). There were no areas of significantly increased cortical thickness in patients with CUD compared to HC. The overlap of decreased cortical thickness from the ROI-based and vertex-wise GLM approach in patients with CUD compared to HC is presented in Supplementary Figure S1.

#### 3.2.2. Relationship between cortical thickness and behavioral-clinical variables

To test whether the ROI-based cortical thickness in patients with CUD was associated with the behavioral-clinical variables, we performed partial correlation analysis for individual ROI-based cortical thickness in the ROIs that showed a significant group difference (Table 2; Figure 1A). We found no significant (*p* > 0.05) correlations for ROI-based cortical thickness in patients with CUD with behavioral-clinical variables (Supplementary Table S2).

#### 3.2.3. Cortical thickness and ‘dose-effect’

To test whether the ROI-based cortical thickness in patients with CUD was associated with the ‘dose-effect’, we performed partial correlation analyses for individual ROI-based cortical thickness in the ROIs that showed a significant group difference (Table 2; Figure 1A). We found no significant (*p* > 0.05) correlations for ROI-based cortical thickness in patients with CUD with the ‘dose-effect’ (Supplementary Table S3).

### 3.3. BrainAGE

#### 3.3.1. Brainage is significantly increased in patients with CUD

**Figure 2:**
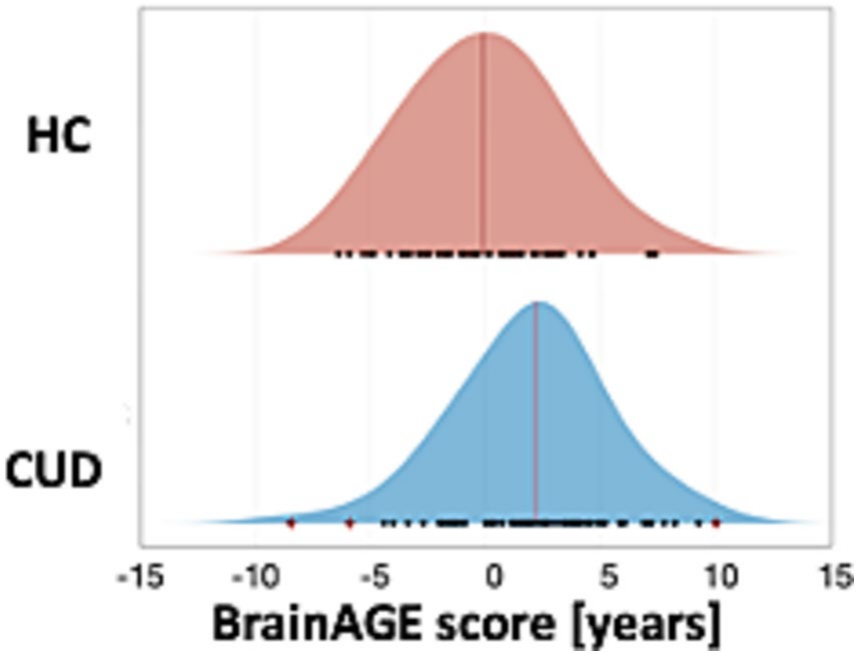
Significantly increased BrainAGE scores in patients with CUD. The results of the BrainAGE analysis showed a significantly increased BrainAGE (*p* < 0.001) for patients with CUD [mean (SD) = 1.97 (±3.53)] compared with HC [mean (SD) = 0.00 (±3.27)]. Black dots indicate individual BrainAGE scores of subjects and outliers are marked by a red cross. The mean is indicated by a red vertical line. BrainAGE, brain age gap estimation; CUD, cocaine use disorder; HC, healthy controls.

To assess the effect of CUD on the difference between chronological and biological brain age, we computed BrainAGE separately for patients with CUD [mean (SD) = 1.97 years (±3.53 years)] and HC [mean (SD) = 0.00 years (±3.27 years)]. The difference between the groups was significant (*p* < 0.001, *t* = 3.35) with a moderate effect size (Cohen’s *d* = 0.58) (Figure 2). This result indicates increased biological brain age in patients with CUD.

#### 3.3.2. Relationship between brain measures and behavioral-clinical variables

**Figure 3:**
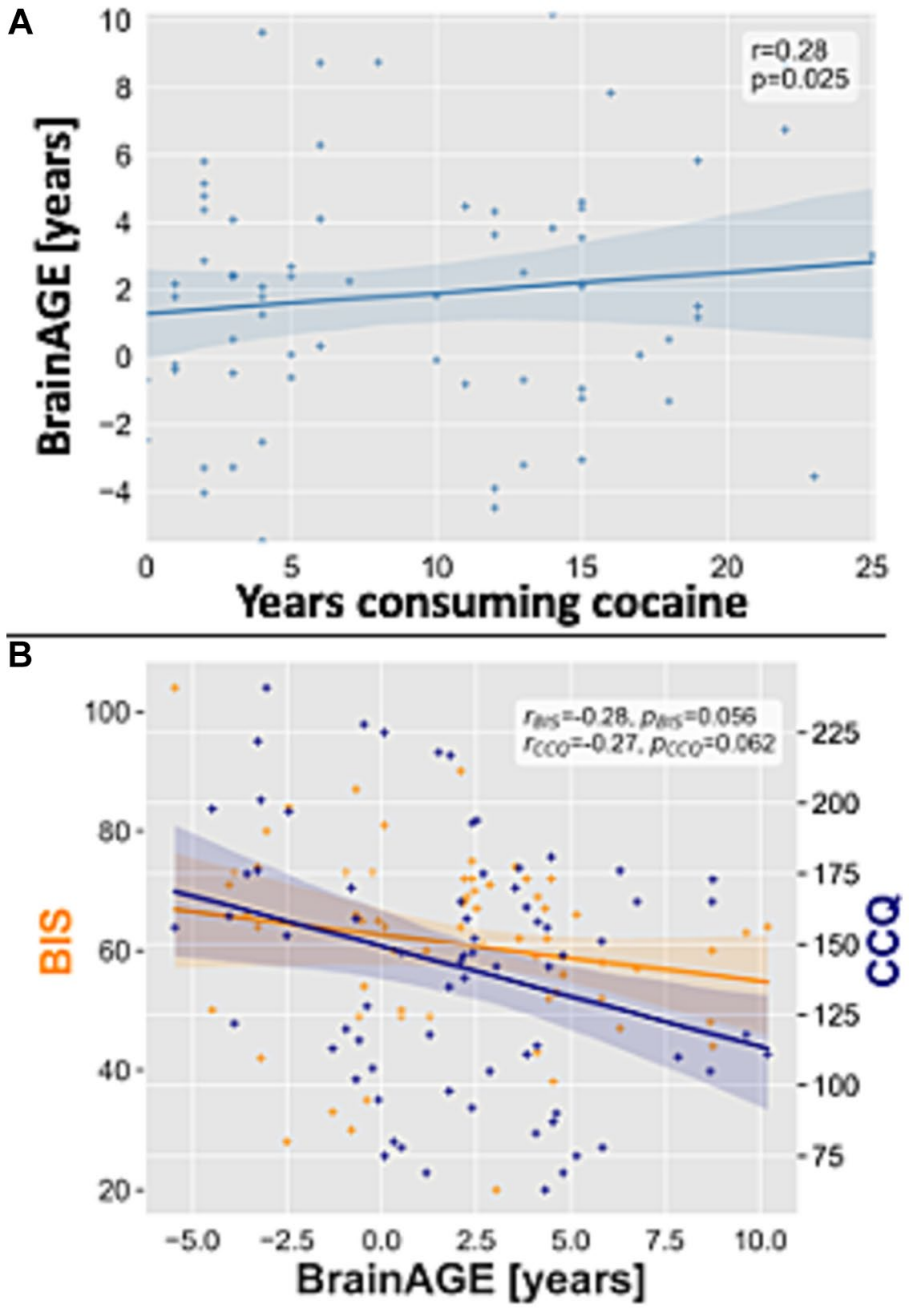
Association between BrainAGE scores in patients with CUD and behavioral-clinical variables and the ‘dose-effect’. **(A)** Scatterplot for BrainAGE scores plotted on the y-axis and the years consuming cocaine plotted on the *x*-axis in patients with CUD. **(B)** Scatterplot for BrainAGE scores plotted on the *x*-axis and the CCQ and BIS scores plotted on the *y*-axis for patients with CUD. Linear regression lines, 90% confidence interval bands, correlation coefficients *r* and value of *p*s were added. BIS, Barratt impulsiveness scale; BrainAGE, brain age gap estimation; CCQ, cocaine craving questionnaire; CUD, cocaine use disorder.

To test whether the BrainAGE scores in patients with CUD were associated with the behavioral-clinical variables, we performed partial correlation analyses for individual BrainAGE scores. We found a non-significant (*p* > 0.05) negative correlation for BrainAGE scores in patients with CUD with the behavioral-clinical variables (Table 3; Figure 3A).

**Table 3:** Relationship between individual BrainAGE scores of patients with CUD and behavioral-clinical variables and the ‘dose-effect’.

*r*-values (*p*-values)	BCST	CCQ	BIS	Avg. weekly CU	Years consuming
BrainAGE score	−0.20 (*p* = 0.173)	−0.27 (*p* = 0.062)	−0.28 (*p* = 0.056)	−0.02 (*p* = 0.455)	0.28 (*p* = 0.025)

Two-tailed partial correlation analyses for the CUD cohort between individual BrainAGE scores and behavioral-clinical variables and one-tailed partial spearman correlation analysis for the CUD cohort between individual BrainAGE scores and the ‘dose-effect’. Sex and age at scan were included as covariates of no interest. Individual correlation coefficients *r* and respective value of *p*s are presented in the table. Bold letters indicate statistical significance defined as *p* < 0.05. Avg., average; BCST, Berg’s card sorting test; BIS, Barratt impulsiveness scale; BrainAGE, brain age gap estimation; CCQ, cocaine craving questionnaire; CU(D), cocaine use (disorder).

#### 3.3.3. BrainAGE and ‘dose-effect’

To test whether the BrainAGE scores in patients with CUD were associated with the ‘dose-effect’, we performed partial correlation analyses for individual BrainAGE scores. We found a significant positive correlation between the years of consuming cocaine and BrainAGE scores (*r* = 0.28, *p* = 0.025, Figure 3B) and no significant correlation with the average weekly cocaine dose (Table 3).

## 4. Discussion

Using the BrainAGE method and surface-based morphometry, we demonstrated a significant decrease in cortical thickness in widespread bilateral association cortices and an increase in BrainAGE in a relatively large cohort of patients with CUD. While lower cognitive performance in patients with CUD was not significantly correlated with structural brain alterations, higher individual BrainAGE scores were significantly correlated with the number of years of cocaine consumption, underlining an association with *prolonged* cocaine use. Our study adds to previous evidence of widespread structural changes in CUD while suggesting a complex interaction between structural, behavioral, and cognitive measures.

The first main finding of our study is an increased BrainAGE score in patients with CUD, indicating accelerated biological brain aging. In an attempt to define the complexity of aging, geroscientific research specifies the key factors of aging as follows: epigenetics, inflammation, macromolecular damage, metabolism, regeneration, and stress (48). These key factors of aging can vary widely and therefore lead to an individual gap in biological and chronological brain age. This offset has been demonstrated in various diseases, such as neurodegenerative, inflammatory, or neurodevelopmental disorders (28–30). In CUD, the individual factors of accelerated aging are not known, but several of those mentioned in geroscience come into question. The results we have shown demonstrate an acceleration of brain aging that exceeds the offset observed in prematurity (28) or stable mild cognitive impairment (49) and is comparable to schizophrenia (50) but below Alzheimer’s disease (26). Another frequently used framework for age-related brain alterations is allostatic load (51). It refers to structural and functional brain changes induced by increased chronic stress that triggers a complex cascade of adaptive physiological processes leading to brain atrophy and cognitive decline (52, 53). There is evidence of allostatic load in CUD, with studies linking chronic cocaine use with increased allostatic load (54). Indeed, the association we demonstrated between BrainAGE scores and the ‘dose-effect’ supports the assumption of causality for increased biological brain aging due to prolonged cocaine use as depicted by years of cocaine use rather than the average weekly intake of cocaine. A similar relationship between years of cocaine use and structural brain changes, i.e., pulvinar volume, has been previously shown (9). While there is evidence for the ‘dose-effect’ in others and our study, the relationship between increased biological brain aging in CUD and behavioral-clinical alterations remains complex. While a decline in impulsivity with higher age has been previously described in healthy individuals (55, 56) and cocaine users (57), we found an additional correlation between increased BrainAGE scores and lower impulsivity after controlling for age, although this was not significant.

The second main finding of our study is the widespread, bilateral decreased cortical thickness in association cortices, mainly in the prefrontal cortices, temporoparietal junction, and posterior cingulate cortex in patients with CUD. The prefrontal cortex is a highly developed brain structure that is mostly unique to primates and thus difficult to study (for a recent review see Carlén) (58). Its basic physiology supports a variety of functions, including cognition, emotional, motivational, social, and perceptual processes (58). This basic functioning modulated by the prefrontal cortex overlaps with functions that are negatively affected in patients with CUD. Notably, decision-making, impulsivity, and attention, which are mainly attributed to the prefrontal cortex, may be involved in the addiction-related processes of patients with CUD (5, 6, 58). A loss of gray matter volume or, more precisely, a reduction in cortical thickness, particularly in prefrontal cortices, in chronic cocaine use has been previously demonstrated and associated with behavioral and cognitive impairments (14–16, 18, 19). In addition, PET-imaging confirmed decreased activity in the orbitofrontal cortex of cocaine users (59).

Recently, a rodent study demonstrated that abnormal neuronal projections to frontal cortices mediated cocaine reward context association, potentially providing a link between structural brain alterations, e.g., reduced prefrontal cortical thickness and accelerated brain aging, and behavioral patterns in CUD (60). Although this study has limitations, particularly with regard to the transferability of prefrontal cortex alterations observed in rodents to those in humans, future studies of cortical brain structure in CUD should focus on white matter alterations to potentially link cortical alterations and functional deficits.

Apart from prefrontal structural brain alterations, we found decreased cortical thickness in the temporoparietal junction and posterior cingulate cortices. These regions are part of the multimodal, widespread association cortex involved in information integration and higher cognition (for a recent review see Sydnor et al.) (61). Interestingly, these cortical regions have an extended multi-decade duration of brain development, thus potentially being more vulnerable to negative effects, e.g., cocaine consumption, which begins in the first three decades (61). Another implication of these structural alterations, particularly in the temporoparietal junction, is their importance in neurodegenerative diseases (62, 63). Further studies are needed to investigate the effects of CUD on aging and potential accelerated neurodegeneration.

While we investigated cortical thickness and BrainAGE in CUD separately, it is important to recognize that these two measures are not independent. Previous research has demonstrated that cortical thickness age-dependently declines across the adult life span (64–67). Notably, both GM volume and cortical thickness are key features in many brain age models (25, 68), and cortical thickness has shown some of the largest disparities in brain age across different durations of illnesses (69). Consequently, the regions with reduced cortical thickness identified in our study might be the main drivers of accelerated brain aging in individuals with CUD. This, in turn, could influence forebrain volume losses in CUD. It is hypothesized that the deterioration of cortical thickness is a driving factor behind reductions in brain volume (69, 70). However, it is important to note that these are speculative assumptions, as several other factors such as cortical surface area, gray/white matter intensity contrast, and curvature may collectively contribute to changes in brain volume (69, 70). Further investigations are needed to comprehensively understand the complex interplay of these factors.

## 5. Limitations

While the relatively large sample size (74 patients with CUD and 62 HC) enhances the generalizability of our findings, the current sample is biased toward patients with CUD with less severe sequelae, as individuals with stronger craving and more cognitive deficits were less likely to participate in an MRI scan or continuation in the study’s clinical and cognitive assessments. Additionally, the original cohort excluded subjects older than 50 years (31). Therefore, our results are rather conservative estimates, as age differences might be larger at older ages, which needs further investigation. We found a significantly accelerated brain aging in patients with CUD, but no significant correlation with the participants’ cognitive or clinical variables. This might indicate a compensatory mechanism between altered structure and functional outcome or be due to the less affected, younger cohort. In addition, while the BCST is a well-established test for the assessment of problem-solving, it may fall short regarding the individuals’ cognitive capacities. Other cognition sensitive tests, like the full-scale IQ test, might have shown an association between decreased cortical thickness or increased BrainAGE with cognitive variables.

## 6. Conclusion

CUD is associated with widespread reductions of cortical thickness, mostly in prefrontal and parietal association cortices, and with dose-dependent, increased brain aging. Further studies are needed to investigate the functional implications of these findings, especially regarding age-related disorders and neurodegeneration.

## Patient consent statement

Written informed consent was obtained from all participants.

## Data availability statement

Publicly available datasets were analyzed in this study. This data can be found here: https://openneuro.org/datasets/ds003346/versions/1.1.2.

## Ethics statement

The studies involving humans were approved by the local ethics committees of the Instituto Nacional de Psiquiatría “Ramón de la Fuente Muñiz” in Mexico City, Mexico. Participants were invited through posters placed in addiction treatment centers. The studies were conducted in accordance with the local legislation and institutional requirements. The participants provided their written informed consent to participate in this study.

## Author contributions

DS: Conceptualization, Data curation, Formal analysis, Funding acquisition, Investigation, Methodology, Project administration, Resources, Software, Supervision, Validation, Visualization, Writing – original draft, Writing – review & editing. BS-K: Writing – review & editing. MT: Writing – review & editing. VS: Writing – review & editing. JS: Writing – review & editing. CZ: Writing – review & editing. CS: Conceptualization, Formal analysis, Methodology, Writing – review & editing. CG: Formal analysis, Investigation, Methodology, Software, Writing – review & editing. DH: Investigation, Methodology, Supervision, Writing – review & editing. TT: Investigation, Methodology, Writing – review & editing.

## Footnotes

^1^ https://www.mathworks.com/products/matlab.html

^2^ http://www.fil.ion.ucl.ac.uk/spm

^3^ https://neuro-jena.github.com/cat

^4^ https://brain-development.org/ixi-dataset
