# Supplementary material for: Lower cortical thickness and increased brain aging in adults with cocaine use disorder
Source: Front Psychiatry. 2023 Nov 13;14:1266770. doi: 10.3389/fpsyt.2023.1266770 (PMC10679447; doi:10.3389/fpsyt.2023.1266770)
Supplement: Supplementary file 1 [file Data_Sheet_1.docx]

**Supplementary Material for:**

**Lower cortical thickness and increased brain aging in adults with cocaine use disorder**

By David Schinz et al.

**Supplement Material**

**Participants**

Eliminated participants as adapted from: The Mexican magnetic resonance imaging dataset of patients with cocaine use disorder: SUDMEX_CONN^28^

**ID Elimination criterion**

37 No MRI study.

39 Diagnosis of Diabetes Mellitus (DM).

63 No cognitive/clinical data.

69 Diagnosis of Diabetes Mellitus (DM).

88 No cognitive/clinical data.

102 Diagnosis of Diabetes Mellitus (DM) and hypertension (HPN) and no

cognitive/clinical data.

119 Diagnosis of hypertension (HPN) and no cognitive/clinical data.

133 Missing clinical data.

134 Missing clinical data.

Abbreviation: ID, identity document

**Surface-based morphometry**

The surface region of interest (ROI) analysis was based on the 70 ROIs defined by the Desikan-Killiany Atlas^1^ included in CAT12:

**Table S1.** ROIs of the Desikan-Killiany Atlas as included in CAT12

| **Region** | **ROI** |
| --- | --- |
| **Left hemisphere**   1. lbankssts 2. lcaudalanteriorcingulate 3. lcaudalmiddlefrontal 4. lcorpuscallosum 5. lcuneus 6. lentorhinal 7. lfusiform 8. linferiorparietal 9. linferiortemporal 10. listhmuscingulate 11. llateraloccipital 12. llateralorbitofrontal 13. llingual 14. lmedialorbitofrontal 15. lmiddletemporal 16. lparahippocampal 17. lparacentral 18. lparsopercularis 19. lparsorbitalis 20. lparstriangularis 21. lpericalcarine 22. lpostcentral 23. lposteriorcingulate 24. lprecentral 25. lprecuneus 26. lrostralanteriorcingulate 27. lrostralmiddlefrontal 28. lsuperiorfrontal 29. lsuperiorparietal 30. lsuperiortemporal 31. lsupramarginal 32. lfrontalpole 33. ltemporalpole 34. ltransversetemporal 35. linsula   **Right hemisphere**   1. rbankssts 2. rcaudalanteriorcingulate 3. rcaudalmiddlefrontal 4. rcorpuscallosum 5. rcuneus 6. rentorhinal 7. rfusiform 8. rinferiorparietal 9. rinferiortemporal 10. risthmuscingulate 11. rlateraloccipital 12. rlateralorbitofrontal 13. rlingual 14. rmedialorbitofrontal 15. rmiddletemporal 16. rparahippocampal 17. rparacentral 18. rparsopercularis 19. rparsorbitalis 20. rparstriangularis 21. rpericalcarine 22. rpostcentral 23. rposteriorcingulate 24. rprecentral 25. rprecuneus 26. rrostralanteriorcingulate 27. rrostralmiddlefrontal 28. rsuperiorfrontal 29. rsuperiorparietal 30. rsuperiortemporal 31. rsupramarginal 32. rfrontalpole 33. rtemporalpole 34. rtransversetemporal 35. rinsula | Banks superior temporal sulcus  Caudal anterior-cingulate cortex  Caudal middle frontal gyrus  Corpus callosum (unmeasured)  Cuneus cortex  Entorhinal cortex  Fusiform gyrus  Inferior parietal cortex  Inferior temporal gyrus  Isthmus-cingulate cortex  Lateral occipital cortex  Lateral orbital frontal cortex  Lingual gyrus  Medial orbital frontal cortex  Middle temporal gyrus  Parahippocampal gyrus  Paracentral lobule  Pars opercularis  Pars orbitalis  Pars triangularis  Pericalcarine cortex  Postcentral gyrus  Posterior-cingulate cortex  Precentral gyrus  Precuneus cortex  Rostral anterior cingulate cortex  Rostral middle frontal gyrus  Superior frontal gyrus  Superior parietal cortex  Superior temporal gyrus  Supramarginal gyrus  Frontal pole  Temporal pole  Transverse temporal gyrus  Insula  Banks superior temporal sulcus  Caudal anterior-cingulate cortex  Caudal middle frontal gyrus  Corpus callosum (unmeasured)  Cuneus cortex  Entorhinal cortex  Fusiform gyrus  Inferior parietal cortex  Inferior temporal gyrus  Isthmus-cingulate cortex  Lateral occipital cortex  Lateral orbitofrontal cortex  Lingual gyrus  Medial orbital frontal cortex  Middle temporal gyrus  Parahippocampal gyrus  Paracentral lobule  Pars opercularis  Pars orbitalis  Pars triangularis  Pericalcarine cortex  Postcentral gyrus  Posterior-cingulate cortex  Precentral gyrus  Precuneus cortex  Rostral anterior cingulate cortex  Rostral middle frontal gyrus  Superior frontal gyrus  Superior parietal cortex  Superior temporal gyrus  Supramarginal gyrus  Frontal pole  Temporal pole  Transverse temporal gyrus  Insula |

**Supplement Results**

**Correlation Analyses for cortical thickness and behavioral-clinical variables and the 'dose-effect'**

The results for the two-tailed partial Spearman correlation analyses between cortical thickness and the behavioral-clinical variables can be found in Table S2. The results for the one-tailed partial Spearman correlation analyses between cortical thickness and the 'dose-effect' can be found in Table S3.

**Table S2.** Relationship between ROI-based cortical thickness and the behavioral-clinical variables in patients with CUD.

| ***p-values*** | **BCST** | **BIS** | **CCQ** |
| --- | --- | --- | --- |
| **ROI** |  |  |  |
| **Left hemisphere** |  |  |  |
| Lateral occipital cortex | 0.48 | 0.05 | 0.21 |
| Superior frontal gyrus | 0.85 | 0.79 | 0.99 |
| Rostral middle frontal gyrus | 0.69 | 0.97 | 0.83 |
| Precuneus cortex | 0.51 | 0.15 | 0.14 |
| Pars triangularis | 0.57 | 0.30 | 0.63 |
| Inferior parietal cortex | 0.57 | 0.66 | 0.93 |
| Caudal middle frontal gyrus | 0.62 | 0.12 | 0.38 |
| **Right hemisphere** |  |  |  |
| Rostral middle frontal gyrus | 0.99 | 0.78 | 0.44 |
| Inferior parietal cortex | 0.52 | 0.36 | 0.24 |
| Cuneus cortex | 0.70 | 0.62 | 0.53 |
| Superior frontal gyrus | 0.72 | 0.87 | 0.94 |
| Precuneus cortex | 0.79 | 0.46 | 0.33 |
| Lingual gyrus | 0.62 | 0.12 | 0.20 |
| Precentral gyrus | 0.39 | 0.69 | 0.48 |
| Banks superior temporal sulcus | 0.69 | 0.44 | 0.22 |

Two-tailed partial correlation analysis for the CUD cohort between ROI-based cortical thickness and behavioral-clinical variables. “Sex” and “age at scan” were included as covariates-of-no-interest. Statistical significance was set at p<0.05.

Abbreviations: BCST, Berg’s Card Sorting Test; CCQ, Cocaine Craving Questionnaire; CUD, cocaine use disorder; ROI, region of interest.

**Table S3.** Relationship between ROI-based cortical thickness and the 'dose-effect' in patients with CUD.

| ***p-values*** | **Avg weekly CU** | **Years consuming** |
| --- | --- | --- |
| **ROI** |  |  |
| **Left hemisphere** |  |  |
| Lateral occipital cortex | 0.39 | 0.35 |
| Superior frontal gyrus | 0.23 | 0.41 |
| Rostral middle frontal gyrus | 0.09 | 0.21 |
| Precuneus cortex | 0.34 | 0.49 |
| Pars triangularis | 0.46 | 0.29 |
| Inferior parietal cortex | 0.36 | 0.40 |
| Caudal middle frontal gyrus | 0.33 | 0.27 |
| **Right hemisphere** |  |  |
| Rostral middle frontal gyrus | 0.44 | 0.47 |
| Inferior parietal cortex | 0.45 | 0.47 |
| Cuneus cortex | 0.44 | 0.47 |
| Superior frontal gyrus | 0.44 | 0.47 |
| Precuneus cortex | 0.45 | 0.47 |
| Lingual gyrus | 0.44 | 0.48 |
| Precentral gyrus | 0.44 | 0.47 |
| Banks superior temporal sulcus | 0.45 | 0.48 |

One-tailed partial correlation analysis for the CUD cohort for ROI-based cortical thickness and the 'dose-effect'. “Sex” and “age at scan” were included as covariates-of-no-interest. Statistical significance was set at p<0.05.

Abbreviations: Avg, average; CU(D), cocaine use (disorder); ROI, region of interest.

**Figure S1. Overlap of decreased cortical thickness from the ROI-based and vertex-wise approach in CUD.**


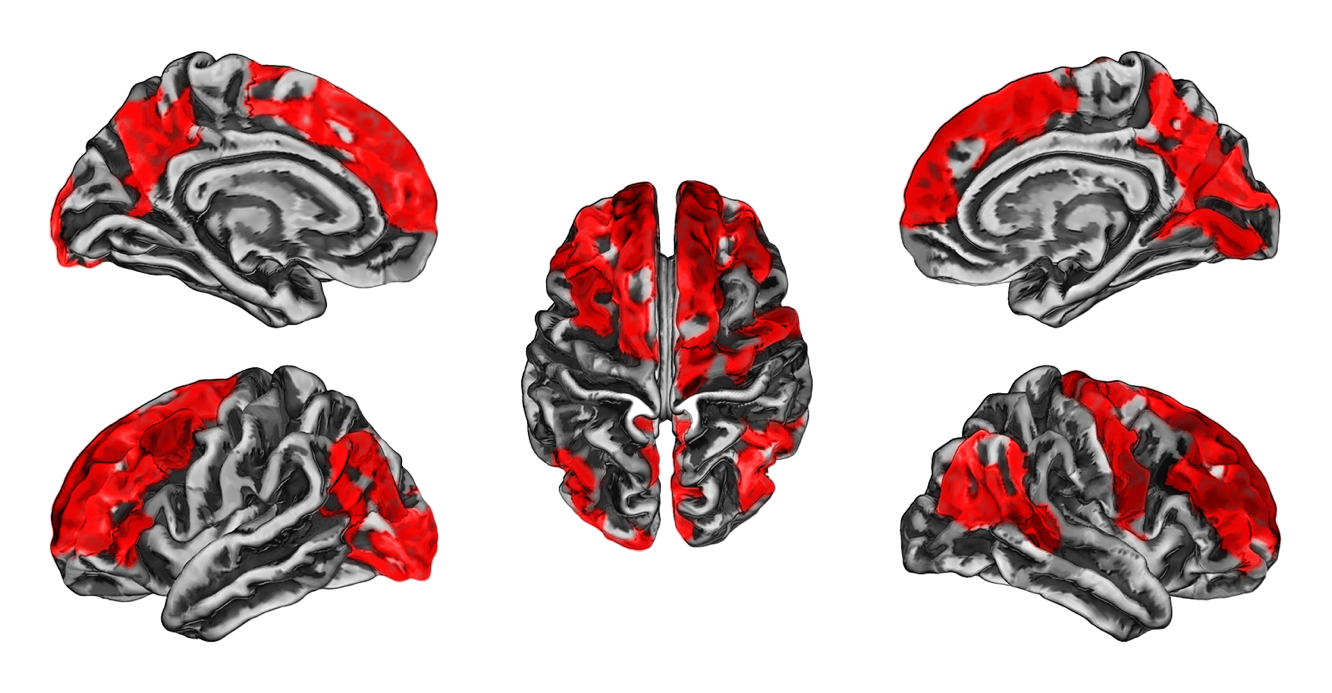


Results of ROI-based and vertex-wise comparison of cortical thickness in patients with CUD compared to HC shows an overlap of 70.05% (highlighted in red) of widespread bilateral reductions in cortical thickness in the prefrontal cortices (superior frontal gyrus and rostral middle frontal gyrus), posterior cingulate cortex, and temporoparietal junction in the CUD cohort. Statistical significance was set at *p*<0.05, FDRc for the ROI-based approach and *p*<0.05, FWEc for the vertex-wise approach.
